# The novel ZEB1-upregulated protein PRTG induced by *Helicobacter pylori* infection promotes gastric carcinogenesis through the cGMP/PKG signaling pathway

**DOI:** 10.1038/s41419-021-03440-1

**Published:** 2021-02-04

**Authors:** Tian Xiang, Chunhui Yuan, Xia Guo, Honghao Wang, Qinzhen Cai, Yun Xiang, Wei Luo, Gao Liu

**Affiliations:** 1 Department of Laboratory Medicine, Central Hospital of Enshi Autonomous Prefecture, Enshi Clinical College, Medical School of Hubei Minzu University, 445000 Enshi, Hubei People’s Republic of China; 2 Department of Laboratory Medicine, Wuhan Medical and Health Center for Women and Children, Tongji Medical College, Huazhong University of Science and Technology, 430016 Wuhan, People’s Republic of China; 3 Department of Gastrointestinal Surgery, Central Hospital of Enshi Autonomous Prefecture, Enshi Clinical College, Medical School of Hubei Minzu University, 445000 Enshi, Hubei People’s Republic of China; 4 Department of Clinical Laboratory, Tianjin Medical University General Hospital, 300052 Tianjin, People’s Republic of China

**Keywords:** Gastric cancer, Oncogenesis

## Abstract

*Helicobacter pylori* (*H. pylori*) is listed as a class I carcinogen in human gastric cancer; however, the underlying mechanisms are poorly understood. In this study, we identified Protogenin (PRTG) was upregulated in both gastric cancer tissues and *H. pylori*-infected tissues by analyzing dysregulated genes in TCGA and GEO databases. Importantly, upregulated PRTG predicted poor prognosis of gastric cancer patients and integrative analysis revealed that PRTG served as an oncogenic protein in gastric cancer and was required for *H. pylori*-mediated tumorigenic activities in in vitro cellular and in vivo tumor-bearing mouse models. Mechanistically, *H. pylori* infection enhanced PRTG expression by promoting transcriptional factor ZEB1 stabilization and recruitment to the PRTG promoter, and which then activated the sub-following cGMP/PKG signaling pathway in bioinformatic and cellular studies. Cellular studies further confirmed that PRTG depended on activating cGMP/PKG axis to promote proliferation, metastasis, and chemoresistance of gastric cancer cells. The PKG inhibitor KT5823 played synergistic anti-tumor effects with cisplatin and paclitaxel to gastric cancer cells in in vitro cellular and in vivo tumor-bearing mouse models. Taken together, our findings suggested that *H. pylori* infection depends on ZEB1 to induce PRTG upregulation, and which leading to the development and progression of gastric cancer through activating cGMP/PKG signaling pathway. Blocking PRTG/cGMP/PKG axis, therefore, presents a promising novel therapeutic strategy for gastric cancer.

## Introduction

According to the World Cancer Research Report, gastric cancer remains the fifth most commonly diagnosed cancer and the third leading cause of cancer-related deaths^1^. Infection with *Helicobacter pylori* (*H. pylori*), a bacterial carcinogen, contributed to ~75% of the global gastric cancer burden and *its* eradication therapy during early stages of the precancerous cascade can prevent gastric cancer development^2^. Recently, a multicenter study further confirmed that post-operative S-1 (oral fluoropyrimidine comprised of tegafur, gimeracil, and oteracil) adjuvant chemotherapy led to an increase in both overall survival and disease-free survival in *H. pylori*-positive advanced gastric cancer patients than *H. pylori*-negative patients^3^. Thus, unveiling the precise mechanisms that regulate cancer development in response to *H. pylori* infection is urgently required for improving the prognosis of gastric cancer patients.

β-catenin, P53 and NF-κB are well-recognized critical targets involved in *H. pylori*-induced dysregulation of anti-tumor/oncogenic signaling pathways and genetic instability in gastric cancer cells, as well as chronic inflammation in epithelial microenvironment^4^. Within gastric epithelial cells, infection with *H. pylori* induces nuclear accumulation of β-catenin and thus enhances the subsequent gastric cancer-initiating cell properties and metastatic potential^4^. Osteopontin expression and downregulation of miR-320a and miR-4496 are required for *H. pylori*-dependent accumulation of β-catenin^5,6^. Genetic instability is emerging hallmark of cancer development and results from direct DNA damage and repair failure. *H. pylori* targets each of these pathways: (1) induces double-strand DNA breaks (DSBs) in an NF-κB dependent manner by enhancing TRAF6-mediated ubiquitination of TAK1; (2) impairs DNA repair functions by promoting p53 degradation, and which is mediated by inhibiting USF1 expression and the following formation of USF1/p53 nuclear complex^7,8^. In addition, *H. pylori-*induced NF-κB activation also promotes proliferation and migration of gastric cancer cells via directly inducing miR-223 and DARPP-32 expression^9,10^. Recently, to further clarify the tumorigenic molecular mechanisms regulated by *H. pylori* infection, integrative analysis of differential messenger RNA (mRNA) expression profiling has been conducted and over 200 differentially expressed mRNAs were identified following infection with different *H. pylori* strains^11^. However, whether these *H. pylori*-dysregulated genes involve in gastric cancer progression remains largely unknown.

In our current study, we identified Protogenin (PRTG) was upregulated in both gastric cancer tissues and *H. pylori*-infected tissues by analyzing dysregulated genes in TCGA and GEO databases. As a member of immunoglobulin domain-containing receptor superfamily, PRTG enhances the migration and survival of cephalic neural crest cells, a transient population of cells that undergo epithelial-to-mesenchymal transition (EMT) along the anteroposterior body axis^12^. We validated the oncogenic roles of PRTG in gastric cancer by activating the downstream cGMP-PKG signaling pathway. Combined PKG inhibitor KT5823 with cisplatin/paclitaxel played synergistic anti-tumor effects in in vitro cellular and in vivo tumor-bearing mouse models. Moreover, molecular studies revealed that *H. pylori* infection enhanced PRTG transcription by promoting EMT transcription factor ZEB1 stabilization and recruitment to the PRTG promoter. Therefore, our findings thus provide new insights into the molecular mechanisms involved in *H. pylori*-mediated gastric cancer progression and suggests that blocking PRTG activation by antagonizing cGMP-PKG signaling through compounds such as KT5823 could provide a novel therapeutic approach to treat gastric cancer.

## Materials and methods

### Gene expression data analysis

The gene expression data used from the NCBI GEO databases (accession numbers GSE29272, GSE5081, GSE62254) and The Cancer Genome Atlas (TCGA) are publicly available. All of these data were downloaded and processed using BRB array tools for further analysis^13^. The relationship between PRTG expression and overall survival (OS) was analyzed using the Kaplan–Meier database (http://kmplot.com). Hazard ratios with 95% confidence intervals and log-rank *p*-values were calculated. Top 500 genes positively associated with PRTG expression in TCGA gastric cancer dataset and Gene ontology enrichment analysis for top 8 biological process controlled by differentially expressed genes were analyzed using R2: Genomics Analysis and Visualization Platform (http://r2.amc.nl).

### Patients and tissue samples

One-hundred fifty-two pair of gastric cancer and the corresponding matched adjacent normal tissues (5 cm away from the tumor tissues) were collected from gastric cancer patients undergoing surgery between January 2015 and December 2018 in Central Hospital of Enshi Autonomous Prefecture. Clinicopathologic characteristics of all patients were also collected, and none of these patients had received local, systemic treatments before surgery. Tumor stages were assessed according to the methods recommended by the American Joint Committee on Cancer (AJCC) staging system.

### Cell culture

The immortalized human gastric cell line GES-1 and gastric cancer cell line MGC-803 were cultured in DMEM (Thermo Scientific HyClone, Beijing, China), gastric cancer cell lines AGS, MKN-45, SGC-7901 and BGC-823 were cultured in RPMI-1640 (Thermo Scientific HyClone, Beijing, China). The cell lines were purchased from the China Center for Type Culture Collection (CCTCC, Chinese Academy of Sciences, Shanghai, China). All cell lines were cultured in medium supplemented with 10% fetal bovine serum (FBS) (HyClone Laboratories, Inc.) and 1% penicillin and streptomycin (North China Pharmaceutical Co., Inc., Shijiazhuang, China). The cells were cultured in a humidified atmosphere containing 5% CO_2_ at 37 °C.

### *H. pylori* strain and bacterial infection

The wild-type strain *H. pylori* ATCC43504 used in this study was obtained from the National Institute for Communicable Disease Control and Prevention, Chinese Centers for Disease Control and Prevention (Beijing, China). *H. pylori* was cultured on Columbia agar plates (Difco Laboratories, Detroit, MI, USA) containing 5% sheep blood at 37 °C for 72 h under microaerophilic conditions using an anaerobic box (Mitsubishi Gas Chemical Co., Inc., Tokyo, Japan). The bacteria were harvested and resuspended with phosphate-buffered saline (PBS) and added to the gastric cancer cells at a multiplicity of infection (MOI) of 10:1.

### Lentivirus production and transduction

Human PRTG complementary DNA (cDNA) was cloned into the lentiviral backbone pLVX-mCMV-Puro. The plasmid was transfected with pCAG-HIVgp (RDB04394, Riken, Japan) and pCMV-VSV-G-RSV-Rev (RDB04393, Riken, Japan) into the HEK293T using Lipofectamine 2000 (Invitrogen, Carlsbad, CA, USA). Lentiviral particles in the supernatant were harvested at 72 h after transfection. AGS cells were then infected with pLVX-PRTG or control pLVX lentivirus (MOI = 50) in the presence of 8 μg/mL polybrene. The cells were treated with puromycin (5 μg/mL) for 2 weeks to select the stably transfected cells, GFP-positive cells were selected as pLVX and pLVX-PRTG and then used for subsequent assays.

### Luciferase reporter assay

The human PRTG promoter sequences (−1625/+107) were obtained by PCR from human genomic DNA. Mutagenesis of the ZEB1-binding sites (site 1 and site 2) in the PRTG promoter was performed using a QuikChange Site-Directed Mutagenesis kit (Stratagene) in accordance with the manufacturer’s protocol. Then, the wild-type (WT) or mutation of ZEB1-binding sites of PRTG promoter were co-transfected with pcDNA3.1-ZEB1 or pcDNA3.1 empty vector control into AGS cells, respectively. Lysates were prepared at 24 h after transfection, and luciferase activities were determined using the Dual-Luciferase Reporter Assay kit (Promega). The ratio of Renilla luciferase activity normalized into Firefly luciferase activities was calculated.

### Chromatin immunoprecipitation (ChIP) assay

The ChIP assay was performed using Pierce^TM^ Magnetic ChIP Kit (#26157) (Thermo Scientific) in accordance with the manufacturer’s protocol. Briefly, confluent AGS Cells (1 × 10^7^) cells were fixed at room temperature in 1% (v/v) formaldehyde for cross-linking. After sonication using Bioruptor 200, 1% of the soluble chromatin fraction was de-cross-linked by heating at 65 °C overnight and used as input. Antibodies against ZEB1 or normal isotype IgG and lysates were incubated with the remaining chromatin fraction overnight at 4 °C with the presence of 50 μL of protein A/G beads. Beads were washed and protein-DNA complexes eluted (1% SDS, 100 mM NaHCO_3_), then cross-links were reversed by heating. DNA was extracted by using the QiaQuick PCR purification kit (Qiagen, Netherlands) and analyzed by quantitative real-time PCR (qRT-PCR). The ChIP primer sequences were listed in Supplementary Table S1.

### Tumor xenograft model

Female athymic nude mice (aged 6–8 weeks) were purchased from Laboratory Animal Research Center in Hubei Province and maintained according to the animal experimentation guidelines. In all, 2 × 10^6^ cells were collected and suspended in 50 μL of normal saline, then subcutaneously injected to establish tumors. As for PRTG-overexpressing cells, tumor volume of each mouse was monitor for 25 days. Then, mice were sacrificed and xenografts tumors were harvested and weighed. Tumor tissues were processed and sectioned for histological Ki67, E-cadherin and N-cadherin evaluation. For CDDP and KT5823 treated mice, tumor development was allowed for 7 days and then randomly divided into four groups. Mice were then intraperitoneally injected with KT5823 (1 mg/kg) or CDDP (5 mg/kg) once per 2 days. Tumor volume of each mouse was monitored from 7 days (day 1) to 29 days (day 22) after inoculation. Then, mice were sacrificed and xenografts tumors were harvested and weighed.

### Cell counting kit-8 assay

Cells (1500/well) were seeded in 96-well plates in triplicate. Ten microliters of CCK-8 solution (Yeasen, Shanghai, China) was added to each well and incubated at 37 °C for 2 h. Then, the absorbance of the dye solution at 450 nm was measured. The optical densities of the cells were assessed 24 h after incubation.

### Immunohistochemistry

The tissue specimens were fixed and embedded in paraffin to make 4 μm-thick slices. Then, the tissue specimens were deparaffinized in xylene, rehydrated in grade alcohol and washed in distilled water. To block endogenous peroxidase activity, slides were incubated with 3% H_2_O_2_ in methanol. The tissue specimens were incubated with anti-PRTG antibody (Calbiochem, La Jolla, CA,) at 4 °C overnight in a humidified chamber, then incubated with horseradish peroxidase-conjugated secondary antibody for 60 min at room temperature. Then, 3,3’-diaminobenzidine (DAB) staining was performed, and the results were observed under a microscope. Tumors were considered positive, if they present only nuclear staining, with or without cytoplasmic staining.

### EdU cell proliferation assay

EdU cell proliferation assay were performed using BeyoClick™ EdU-647 assay kit (Beyotime, Shanghai, China) according to the manufacturer’s instructions. In brief, the cells (4 × 10^3^) were seeded into 96-well plates and exposed to 10 µM EdU solution for 2 h at 37 °C. Cells were then fixed with 4% paraformaldehyde and permeabilized with 0.5% triton X-100. Next, cells were incubated in the Click Additive Solution for 30 min and stained with DAPI in the dark. Three fields were randomly selected and then imaged using a fluorescence microscope (×200) (Olympus IX73; Olympus Corporation). The percentage of EdU-positive cells was defined as the proliferation rate.

### Immunofluorescence assay

Cells were grown on coverslips, irradiated, washed with PBS, fixed with 4% formaldehyde, and permeabilized with 0.1% NP-40 for 5 min at room temperature. The cells were then incubated in blocking buffer (PBS containing 3% BSA) for 30 min, followed by incubation with the primary antibody (the pH2AX^Ser139^ antibody or the anti-PRTG antibody) and blocking with 1% BSA in PBS for 1 h. Following PBS washing, the cells were incubated with Cy3-conjugated goat anti-mouse secondary Ab (Jackson ImmunoResearch, PA, USA) for 1 h. Following a wash with PBS-containing 4,6-diamidino-2-phenylindole (DAPI; Invitrogen), coverslips were mounted onto glass slides using Permount solution (Fisher Scientific, Pittsburgh, PA, USA). Images were visualized under Olympus IX73 microscope.

### Small-interfering RNA (siRNA) transfection

The silencing siRNA of indicated genes and non-target control siRNA (siNTC) were synthesized from RiboBio (Guangzhou, China). The transfection of siRNAs in AGS cells was conducted with a Lipofectamine 2000 reagent according to manufacturer’s protocol. After reaching 50% ~60% confluence, AGS cells were transfected with 100 nM siRNA. Transfection efficiency was measured by qRT-PCR or western blot 48 h post transfection.

### Cell migration and invasion assays

Briefly, 24-well Transwell plates (pore size, 8 µm; Corning, Inc.) were used for cell invasion and migration assays as we described priviously^14^. For the cell migration assay, cell suspension of 1 × 10^5^ cells in 200 µL serum-free DMEM was seeded into the upper chambers of 24-well plates. The bottom chamber contained 800 µL DMEM supplemented with 10% FBS. After 24 h at 37 °C, the non-migrating cells in the upper surface of the membrane were gently removed with a cotton swab, and the migrated cells in the lower surface of the membrane were washed twice with PBS, fixed with 4% paraformaldehyde for 30 min and stained with 0.1% crystal violet for 5 min. Finally, images were captured and cells were counted with a light inverted microscope. For the Matrigel-based invasion assay, a total of 100 µL Matrigel (BD Biosciences) were pretreated in the wells, and gastric cancer cells (1 × 10^5^ cells per chamber) were added into the upper chamber. A total of 800 µL Dulbecco’s modified Eagle medium (DMEM) supplemented with 10% FBS were added into the lower chamber. The subsequent steps were the same as those conducted for the cell migration assay.

### Western blot

The cells were lysed in a radioimmunoprecipitation assay (RIPA) buffer (Beyotime, Shanghai, China) containing 1 mM phenylmethylsulfonyl fluoride, and the total protein content was measured using a bicinchoninic acid protein assay kit (Beyotime, ShangHai, China). Cells protein lysates was separated on a 12% sodium dodecyl sulfate (SDS)-polyacrylamide gel, and then electroblotted onto a polyvinylidene fluoride (PVDF) membrane. After blocking in Tris buffered saline (10 mM Tris-HCl, pH 8.0, containing 150 mM NaCl) containing 5% (w/v) skim milk powder and 0.5% (v/v) Tween 20, the membrane was cultured with the specific primary antibodies. Bound antibodies were detected with HRP-conjugated secondary antibodies and visualized by chemiluminescence (Pierce ECL Western Blotting Substrate). The following primary antibodies were used: anti-PRTG antibody (CF501394, OriGene), ZEB1(21544-1-AP, Proteintech), E-Cadherin (13-1700, Invitrogen), N-Cadherin (33-3900, Invitrogen), Vimentin (ab92547, abcam), Snail (26183-1-AP, Proteintech), sGC (ab189176, abcam), PKG1 (3248, CST), PKG2 (55138-1-AP, Proteintech), PDE5A (ab28761, abcam), VASP (13472-1-AP, Proteintech), Phospho-VASP^Ser239^ (ab194747, abcam), GAPDH (sc-47724, Santacruz), Caspase-3 (19677-1-AP, Proteintech), p21 (10355-1-AP, Proteintech), Bid (10988-1-AP, Proteintech), BCL2 (12789-1-AP, Proteintech), Phospho-H2AX^Ser139^ (2577, CST), BIRC3 (24304-1-AP, Proteintech), HDAC1 (sc-81598, Santa Cruz), Ubiquitin (10201-2-AP, Proteintech).

### Quantitative real-time PCR (qRT-PCR)

Total RNA extracted from using gastric cancer tissues and cells was treated with Trizol reagent (Invitrogen, CA, USA). Subsequently, complementary DNA was generated from 1 μg total RNA using PrimeScript RT Master Mix (Takara Bio Inc., Kusatsu, Japan). qRT-PCR was conducted using 2 × SYBR green PCR master mix (TaKaRa, Dalian, China) according to the manufacturer’s protocol to detect mRNA levels of indicated genes. The ABI 7300 (Applied Biosystems, CA, USA) was used to perform the amplification reaction. Each experiment was performed in triplicate. Gene expression values were normalized relative to expression of the housekeeping gene GAPDH. The gene-specific primer sequences are listed in Supplementary Table S1.

### Flow cytometry

Apoptosis rates and cell cycle assay were evaluated by flow cytometry (FACSCalibur; BD Biosciences, San Jose, CA, USA). Ten-thousand cells were counted per experiment. For cell apoptosis analysis, cells were incubated with annexin V-FITC and propidium iodide using an Annexin V-FITC Apoptosis Detection Kit (Biolegend) according to the manufacturer’s protocol at 4 °C in the dark for 0.5 h. The fluorescence of the stained cells was then examined by flow cytometry analysis. For cell cycle analysis, cells were incubated with 450 µL propidium iodide (PI) and 50 µL RNase A using the BD Cycletest™ Plus DNA Kit (BD Biosciences, USA) in the dark at room temperature for 1 h, and flow cytometric analysis was carried out.

### Preparation of nuclear and cytoplasmic fractions

Nuclear and cytoplasmic extractions were performed using an NE-PER™ Nuclear Cytoplasmic Extraction Reagent kit (Thermofisher, Shanghai, China) according to the manufacturer’s protocol.

### Statistical analysis

Results were expressed as mean ± SD of at least three independent experiments. Unpaired *t*-test or one-way ANOVA followed by Neuman-Keuls post hoc test was performed for data analysis. Survival analysis was carried out by the Kaplan–Meier method and subjected to the log-rank test. A *p*-value < 0.05 was considered statistically significant. All statistical analyses were performed using GraphPad Prism version 6.00 (GraphPad Software, Inc.).

## Results

### PRTG is negatively associated with the prognosis of gastric cancer

**Fig. 1:**
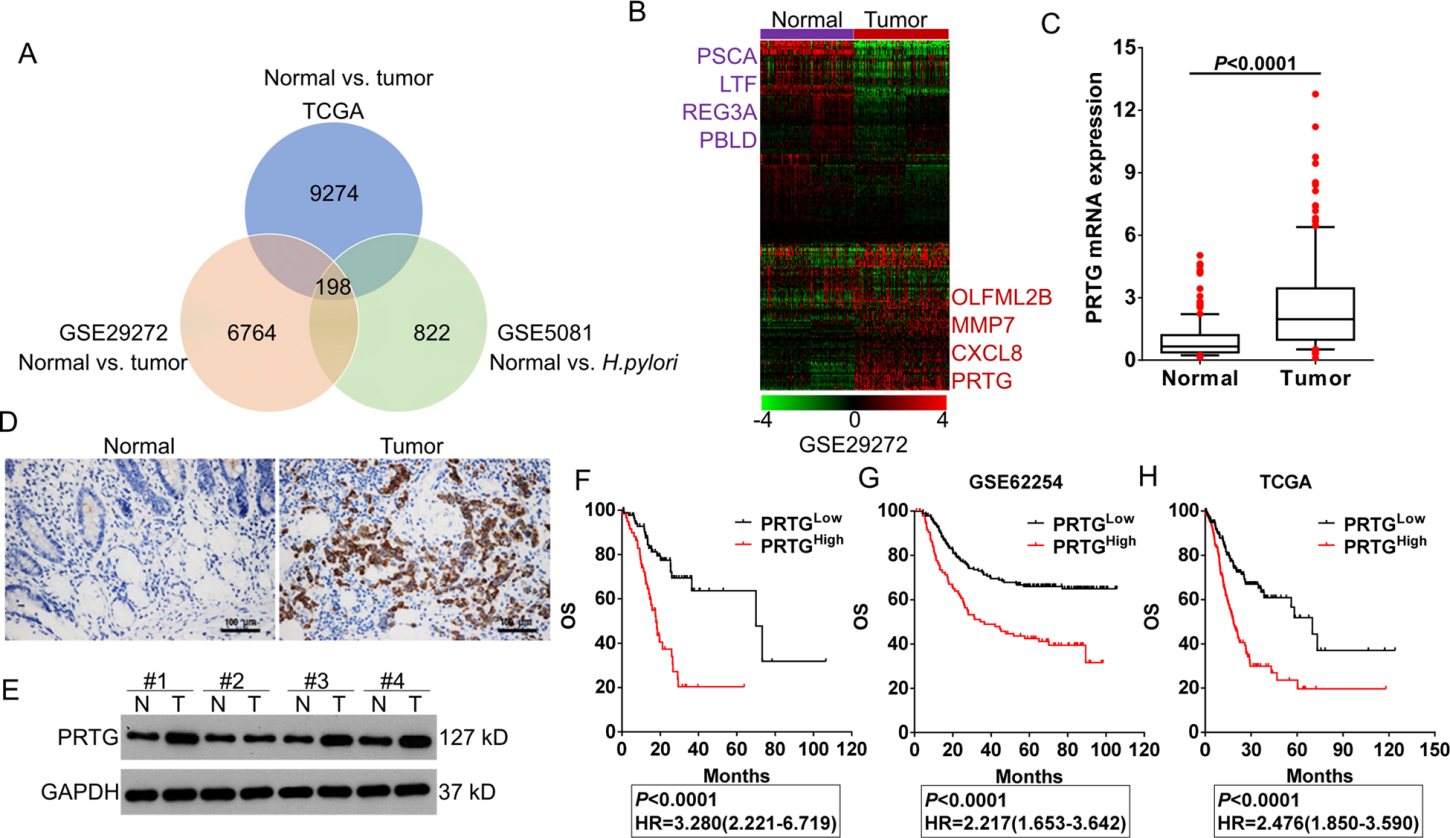
PRTG is upregulated in both gastric cancer and *H. pylori*-infected tissues. **A** Venn diagram of genes showing significant differential expression between normal and cancer tissues in two Gastric cancer patient cohorts (TCGA, GSE29272), as well as differential genes between normal and *H. pylori*-infected tissues in dataset GSE5081. **B** A heat map of the 198 differentially expressed genes of Fig. 1A in the GEO dataset GSE29272. Eight top-ranked genes were highlighted in purple or red text. **C** PRTG expression was examined by qRT-PCR in local 152 human gastric cancer tissues compared with normal tissues. Boxes represent medians and interquartile ranges, with whiskers indicating 10% to 90% range. Dots represent patients who fell outside the 10% to 90% range. **D** IHC was used to detect the expression level of PRTG in gastric cancer and adjacent normal tissues. **E** western blot analysis of the expression level of PRTG in in gastric cancer and adjacent normal tissues. **F**–**H** Kaplan–Meier survival plots analysis of the overall survival for 152 gastric cancer patients in local hospital (**F**), GEO dataset GSE62254 (**G**) and TCGA (**H**) with high or low PRTG expression.

To identify genes involved in gastric cancer caused by *H. pylori* infection, we compared normal gastric samples to tumor samples (TCGA database, GSE29272) and *H. pylori*-infected samples (GSE5081) by applying class comparison analysis^15^. A total of 198 differentially expressed mRNAs that associated with both gastric cancer and *H. pylori* infection were identified with the thresholds of a *p*-value < 0.05 and a | log2 FC | ≥ 1 (Fig. 1A). Strikingly, we found that PRTG, a cell adhesion related protein, was significantly upregulated in gastric cancer tissues and *H. pylori*-infected tissues (Fig. 1A, B).

To confirm the reliability of present bioinformatics analysis, PRTG expression levels were further evaluated in local gastric cancer tissues and cell lines. Consistent with bioinformatics analysis results, the expression levels of PRTG were significantly elevated in gastric cancer tissues (Fig. 1C–E and Fig. S1A) and gastric cancer cell lines (Fig. S1B). We then examined the clinicopathological characteristics of PRTG in gastric cancer patients, the results indicated that PRTG was significantly associated with tumor size, *H. pylori* infection status, lymph node invasion, tumor differentiation, T stage and advanced TNM stage (Table 1). Furthermore, patients with higher PRTG expression presented with worse overall survival in gastric cancer patients from the local hospital (Fig. 1F), GEO dataset GSE62254 (Fig. 1G) and TCGA database (Fig. 1H). Thus, these results indicate that PRTG may play an important role in the progression of gastric cancer caused by *H. pylori* infection via acting as an oncogenic protein.

**Table 1:** Association between PRTG expression and clinicopathologic characteristics of gastric cancer patients from local hospital.

Characteristics	Cases	PRTG mRNA (mean ± SD)	*p*-value
Gender			0.7408
Male	93 (61.2)	2.669 ± 2.515	
Female	59 (38.8)	2.802 ± 2.275	
Age (years)			0.1161
≥60	98 (64.5)	2.491 ± 2.270	
<60	54 (35.5)	3.149 ± 2.660	
Tumor size (longest dimension)			<0.0001
≤4 cm	92 (60.5)	2.066 ± 1.914	
>4 cm	60 (39.5)	3.724 ± 2.761	
*H. pylori* infection			0.0002
No	54 (35.5)	1.751 ± 1.604	
Yes	98 (64.5)	3.255 ± 2.624	
Tumor location			0.9197
Up	23 (15.1)	2.581 ± 2.186	
Middle	74 (48.7)	2.694 ± 2.568	
Down	55 (36.2)	2.815 ± 2.338	
Lauren classification			<0.0001
Intestinal	78 (51.3)	2.016 ± 1.962	
Diffuse	65 (42.8)	3.750 ± 2.689	
Mixed	9 (5.9)	1.392 ± 0.093	
Tumor differentiation			0.0037
G1-G2	49 (32.2)	1.903 ± 1.593	
G3	103 (67.8)	3.110 ± 2.643	
TNM stage			0.0128
I–II	78 (51.3)	2.248 ± 2.169	
III–IV	74 (48.7)	3.219 ± 2.576	
Lymph node/distal metastasis			<0.0001
No	54 (35.5)	1.348 ± 1.000	
Yes	98 (64.5)	3.477 ± 2.632	
T stage			0.0044
T1-2	48 (31.6)	1.905 ± 1.938	
T3-4	104 (68.4)	3.097 ± 2.530	

### PRTG plays an oncogenic role in gastric cancer

**Fig. 2:**
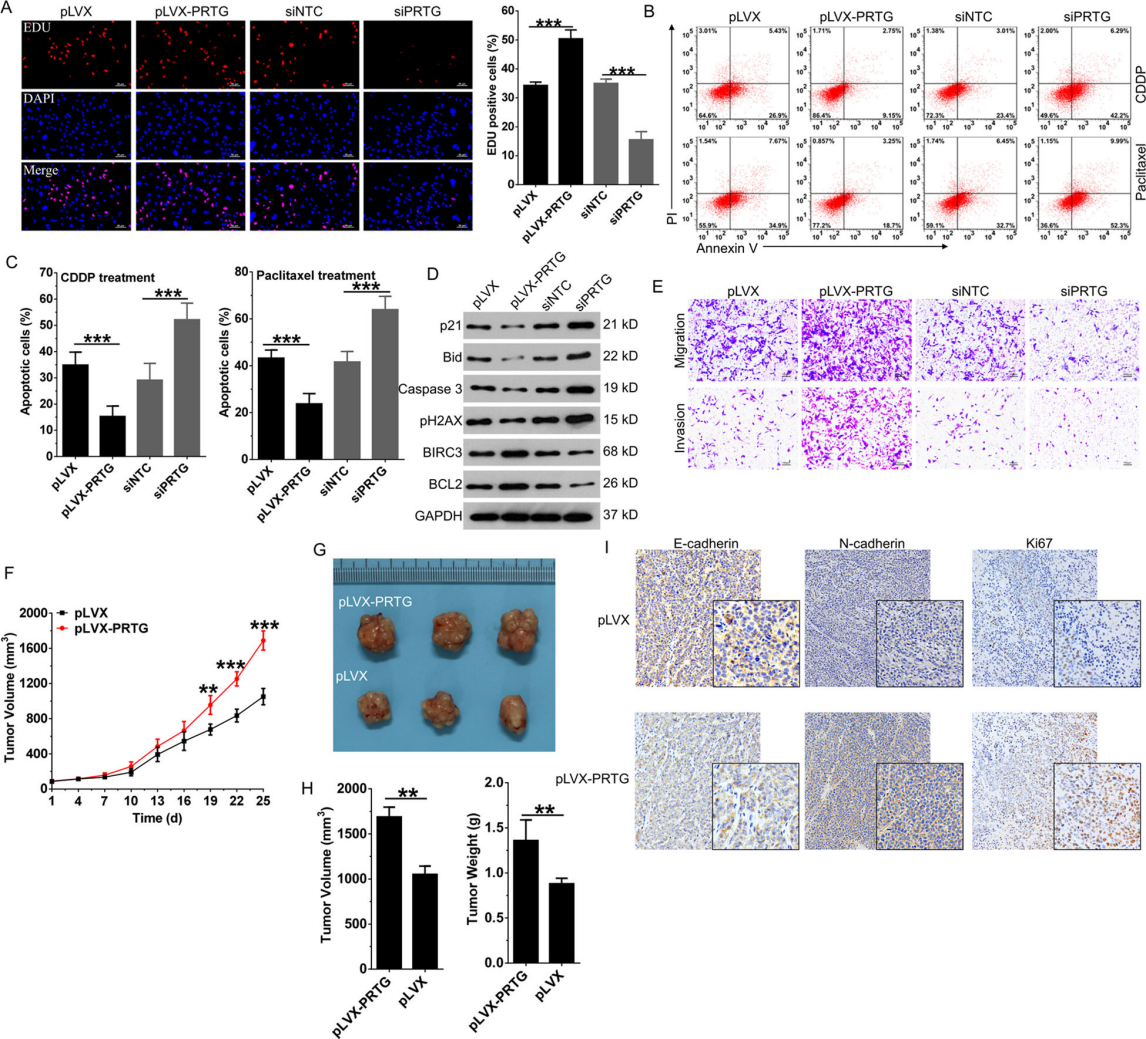
PRTG plays oncogenic activities in gastric cancer progression. **A** EdU assay after PRTG stable overexpression or transient knockdown in AGS cells. **B** Cell apoptosis was detected in PRTG overexpressing or silencing AGS cells after treated with chemotherapy drugs (paclitaxel, 20 nM; CDDP, 5 μM) for 48 h. **C** Statistical analysis of apoptosis in AGS cells (*n* = 3, related to Fig. 2B). **D** Western blot was used to detect the effect of PRTG on the expression of apoptosis-related proteins in paclitaxel-treated AGS cells of **C**. **E** Transwell assay was used to detect the invasion and migration ability of PRTG overexpressing or silencing AGS cells. **F** AGS cells stably expressing empty vector (pLVX) or pLVX-PRTG were injected into the nude mice (*n* = 12) and tumor volumes were monitored. **G** Tumor volumes of 3 mice were presented 25 days post inoculation. **H** Tumor volumes and weights (*n* = 12) were measured 25 days post inoculation. **I** The expression of Ki67, E-cadherin and N-cadherin were detected by IHC. Data were presented as mean ± SD from the three independent replicates. **P < 0.01; ***P < 0.0001. CDDP, cisplatin; pLVX, empty control pLVX lentivirus.

Next, PRTG expression was manipulated in AGS cells (Fig. S1C), which had the highest level of PRTG expression (Fig. S1B) and is known as a low-grade malignant gastric tumor cell line, to identify its biological functions in gastric cancer. EdU assay showed that PRTG overexpression promoted cellular proliferation, whereas PRTG silencing significantly inhibited cell viability in AGS cells (Fig. 2A). Furthermore, PRTG overexpression also inhibited apoptosis induced by cisplatin (CDDP) and paclitaxel, which are chemotherapeutic drugs commonly used systemically in gastric cancer treatment, and PRTG silencing significantly enhanced chemosensitivity of gastric cancer cells (Fig. 2B, C and Fig. S1D). Detection of pro-apoptotic markers such as p21, Bid, caspase-3 and pH2AX, as well as anti-apoptotic markers such as BCL-2 and BIRC3 in paclitaxel-treated cells further confirmed the anti-apoptotic effects of PRTG (Fig. 2D). Cell cycle arrest has been identified as the major mechanism that contributes to enhanced chemosensitivity^16^, PRTG silencing enhanced S phase arrest in CDDP treated cells and G2/M phase arrest in paclitaxel-treated cells (Fig. S1E, 1F). As expected, PRTG silencing increased the formation of pH2AX foci in paclitaxel-treated cells, which represent unrepaired DNA double-strand breaks (DSBs) (Fig. S1G). Transwell assay showed that PRTG significantly promoted migration, invasion (Fig. 2E and S1H) and EMT progression (Fig. S1I) of AGS cells. Then, we further manipulated and evaluated the oncogenic role of PRTG in MGC-803 cells (Fig. S2A), which has the lowest PRTG expression within different gastric cancer cell lines (Fig. S1B). Same as the results obtained in AGS cells, PRTG also significantly promoted proliferation, migration, invasion and chemoresistant potency of MGC-803 cells (Fig. S2B–S2D).

To further validate the oncogenic effects of PRTG in vivo, PRTG-overexpressing cells were subcutaneously injected into nude mice. Tumor growth was significantly accelerated in the pLVX-PRTG group compared to the empty control (pLVX) (Fig. 2F). Meanwhile, the volume and weight of the tumors in the pLVX-PRTG group on day 21 were markedly higher than which in the control group (Fig. 2G, H). Same with in vitro results, we further found that PRTG promoted proliferation and EMT progression in vivo as reflected by Ki67, N-cadherin, and E-cadherin stain (Fig. 2I). Thus, these results indicate that PRTG plays an oncogenic role in gastric cancer.

### PRTG is an important mediator for *H. pylori* to perform tumorigenic activities

**Fig. 3:**
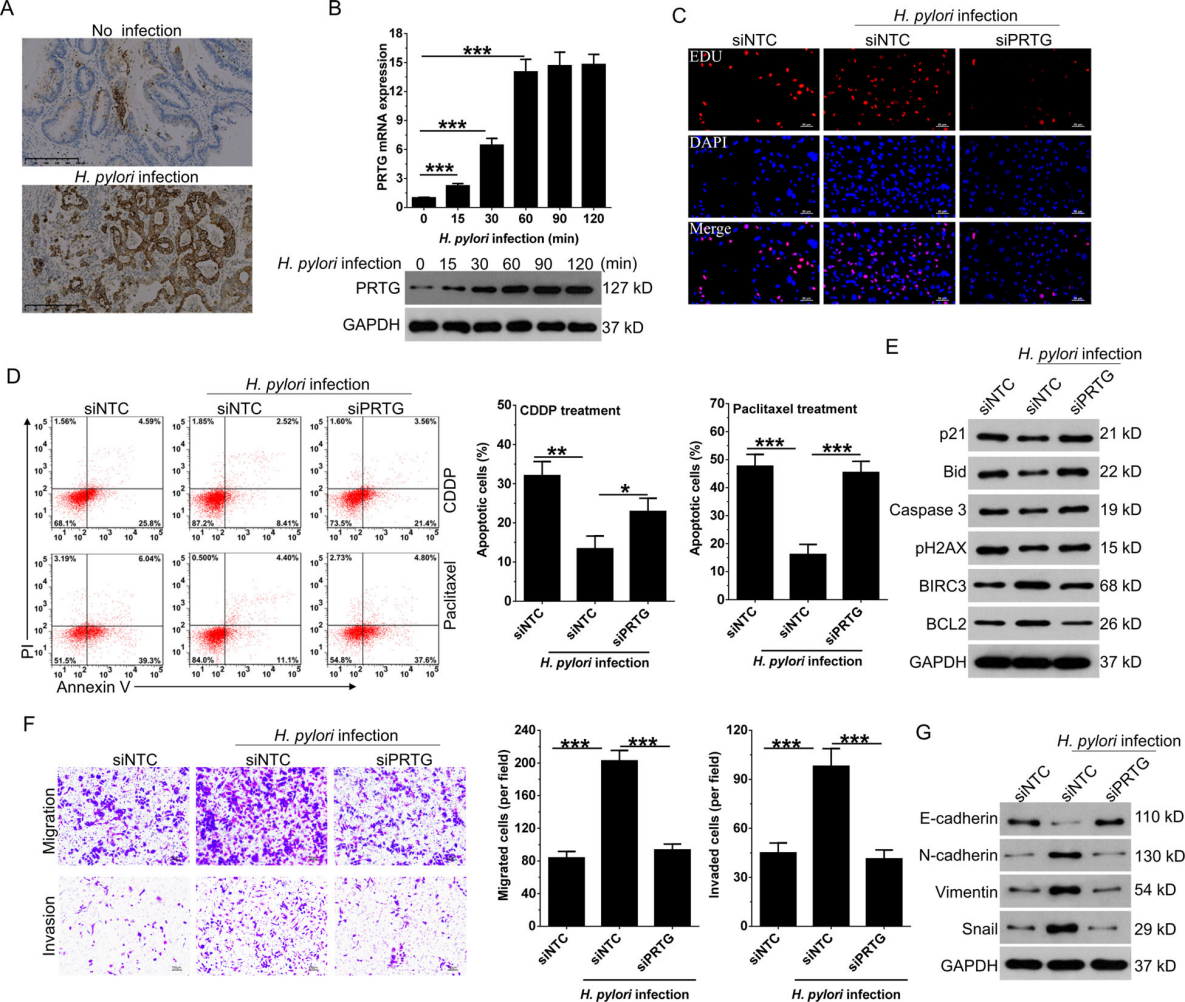
PRTG is required for *H. pylori* to promote GC progression. **A** IHC staining of PRTG expression in GC tissues collected from local hospital with or without *H. pylori* infection. **B** qRT-PCR and western blot analysis of PRTG in AGS cells at different time points post infection with *H. pylori* (MOI = 10:1). **C** EdU assay was used to detect the effect of *H. pylori* infection on the proliferation of AGS cells after silencing PRTG expression. **D** Cell apoptosis was detected in PRTG silencing AGS cells after treated with chemotherapy drugs (paclitaxel, 20 nM; CDDP, 5 μM) and infected with *H. pylori* (MOI = 10:1) for 48 h. **E** Western blot analyses were employed to analyze the expression of apoptosis-related protein in PRTG silencing AGS cells after treated with the paclitaxel and *H. pylori* for 48 h. **F** Transwell assays were used to detect the invasion and migration ability of PRTG silencing AGS cells after infected with *H. pylori* for 48 h. **G** Western blot analyses were used to detect the expression of EMT markers in PRTG silencing AGS cells after infected with *H. pylori* for 48 h. Data were presented as mean ± SD from the three independent replicates. *P < 0.05; **P < 0.01; ***P < 0.0001. CDDP, cisplatin; EMT, epithelial to mesenchymal transition; siNTC, non-target control siRNA.

In clinical setting, PRTG expression was upregulated in *H. pylori*-infected gastric cancer tissues (Figs. 3A, S3A and Table 1). To further confirm the effects of *H. pylori* infection on PRTG expression, AGS cells were infected with *H. pylori* at MOI (multiplicity of infection) of 10:1. Our results showed that PRTG expression was gradually increased in response to *H. pylori* infection in a time-course analysis (Fig. 3B). Furthermore, knockdown of PRTG expression (Fig. S3B) significantly blocked the oncogenic effects of *H. pylori* on gastric cancer, which was reflected by decreased proliferation (Figs. 3C and S3C), increased apoptosis to chemotherapeutic drugs (Fig. 3D, E), G2/M or S phase arrests (Fig. S3D, E) and pH2AX foci formation (Fig. S3F), decreased migration, invasion and EMT progression (Fig. 3F, G) in PRTG silencing cells compared to non-targeting controls. These results indicate that *H. pylori* infection promotes the tumorigenesis of gastric cancer via inducing the expression of PRTG.

### ZEB1 directly upregulates PRTG transcription

**Fig. 4:**
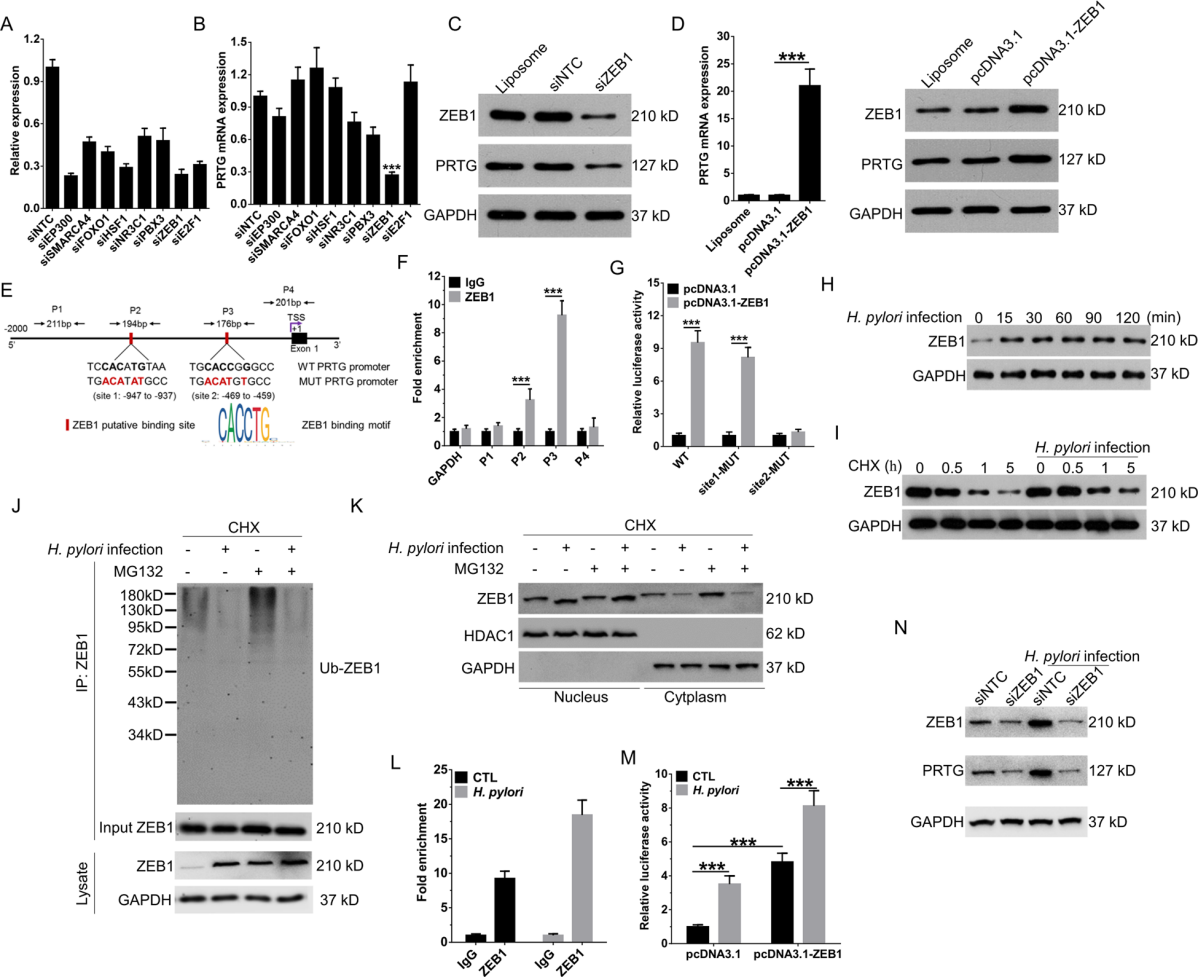
*H. pylori* promotes ZEB1 stabilization and recruitment to PRTG promoter. **A** qRT-PCR was used to detect the silencing efficiency of siRNA targeted to corresponded transcription factor in AGS cells. Relative expression meant the expression of siRNA targeted transcription factor refer to the expression level of cells transfected with control siRNA. **B** The expression of PRTG in AGS cells transfected with indicated siRNA targeted to corresponded transcription factor was detected by qRT-PCR. **C** The expression of ZEB1 and PRTG in ZEB1 silencing AGS cells were detected by western blot. Liposome represented transfection regent control. **D** qRT-PCR and western blot were used to the expression of PRTG and ZEB1 in AGS cells transiently transfected with pcDNA3.1 empty vector or pcDNA3.1-ZEB1. **E** Predicted binding sites of the transcription factor ZEB1 to PRTG promoter (site 1 and site 2). P1 and P4 represented distal regions far from ZEB1 predicted binding sites in PRTG promoter. **F** The binding site of ZEB1 to PRTG promoter was identified by ChIP-qPCR. **G** AGS cells were co-transfected with the ZEB1 expression plasmid and PRTG promoter luciferase reporter constructs. Extract luciferase activities were determined 24 h after transfection. **H** The expression of ZEB1 in AGS cells at different time points post *H. pylori* infection was detected by western blot. **I** The effect of *H. pylori* on the stability of ZEB1 protein in AGS cells treated with CHX (5 μg/mL) for the indicated times was detected by western blot. **J** AGS cells were infected with *H. pylori* and treated with MG132 (10 μM) and CHX for 6 h. whole-cell lysates of uninfected and *H. pylori-*infected cells were immunoprecipitated (IP) with anti-ZEB1 antibody and then immunoblotted for detection of ubiquitinated ZEB1 (Ub-ZEB1). **K** AGS cells were treated as indicated and ZEB1 protein expression in cellular fractions were detected by western blot. **L** ChIP-qPCR analysis of the effect of *H. pylori* infection on the binding of ZEB1 to the PRTG promoter. **M** The luciferase reporter assay was used to detect the effect of *H. pylori* infection on the binding of ZEB1 to the PRTG promoter. **N** Western blot was used to detect the effect of ZEB1 silencing on the *H. pylori*-induced PRTG expression. Data were presented as mean ± SD from the three independent replicates. ***P < 0.0001. CHX, cycloheximide; siNTC, non-target control siRNA.

To acquire the potential transcription factors that regulate PRTG expression, the promoter sequence of PRTG was extracted from bioinformatics programs UCSC Genome Browser and analyzed by Gene Transcription Regulation Database, JASPAR and ChIP-Atlas-Enrichment Analysis. We identified a candidate list of transcriptional regulators of transcription factors (TFs) predicted to bind the PRTG promoter and examined the effect of silencing these TFs on PRTG expression (Fig. 4A, B). Depletion of ZEB1 consistently resulted in about 4-fold reduction of PRTG mRNA level (Fig. 4B) and concomitant loss of protein expression (Fig. 4C). Next, we further confirmed that overexpression of ZEB1 significantly enhanced the expression of PRTG in gastric cancer cells (Fig. 4D). In addition, ZEB1 positively associated PRTG expression (Fig. S4A) and increased (Fig. S4,B) in gastric cancer tissues, as well as predicted poor prognosis of gastric cancer patients (Fig. S4C and S4D).

To determine whether the regulation of PRTG expression by ZEB1 is direct, we assessed the binding of ZEB1 to the PRTG promoter, which is predicted to contain two consensus-binding sites (Fig. 4E). Chromatin immunoprecipitation (ChIP) of ZEB1 antibody followed by qPCR using primers spanning the ZEB1 putative-binding site 1 and site 2 in the PRTG promoter showed about 3-fold and 9-fold enrichment in PRTG signal over ChIP with non-specific IgG, respectively (Fig. 4F). Meanwhile, negative controls, GAPDH and primers spanning PRTG distal regions (P1 and P4) showed no significant enrichment (Fig. 4F). To confirm the ability of ZEB1 to control PRTG expression, we constructed wild-type luciferase reporters vector containing ZEB1 putative-binding site 1 or 2 and mutant luciferase reporter vector in which the ZEB1-binding sites were mutated (Fig. 4E). The AGS cells were co-transfected with the luciferase reporter vector and ZEB1 overexpressing plasmid. The luciferase assay showed that ZEB1 overexpression significantly increased the luciferase activity driven by the wild-type pGL3-PRTG site 2 promoter, whereas ZEB1 overexpression has no significant effect on the luciferase activity driven by mutant site 2, wild-type and mutant site 1 promoter (Fig. 4G). Overall, these results suggest that ZEB1 is involved in PRTG upregulation via binding to the site 2 of PRTG promoter and increasing its expression.

### *H. pylori* infection promotes stabilization and recruitment ZEB1 to PRTG promoter

To explore whether *H. pylori* infection depended on ZEB1 to induce PRTG expression, we firstly detected the expression of ZEB1 in *H. pylori*-infected and non-infected gastric cancer tissues. The results showed that ZEB1 expression significantly increased in *H. pylori*-infected tissues (Fig. S4E). Next, the expression of ZEB1 in response to *H. pylori* infection in gastric cancer cells was then evaluated by western blot and qRT-PCR analysis. Strikingly, we found that ZEB1 mRNA expression showed no change (Fig. S4F and S4G), while protein expression significantly increased in both AGS cells and MGC-803 cells (Fig. 4H and S4H) response to *H. pylori* infection. Thus, we hypothesized that *H. pylori* infection may influence the stability of ZEB1. After pre-treatment with the protein synthesis inhibitor cycloheximide (CHX), ZEB1 protein expression was more slowly degraded in *H. pylori-*infected cells (Fig. 4I). Ubiquitination in nucleus mediates ZEB1 translocation and subsequent proteasomal degradation in cytoplasm^17,18^. With the presence of CHX, ZEB1 ubiquitination and steady-state level were significantly increased in uninfected cells when the proteasome was inhibited with MG132. while *H. pylori* infection significantly decreased ZEB1 ubiquitination and which was almost undetectable even the proteasome was blocked (Fig. 4J). Importantly, the amount of steady-state ZEB1 was significantly increased due to *H. pylori* infection, which indicating that *H. pylori* infection promotes ZEB1 stabilization by inhibiting ubiquitination-mediated proteasomal degradation. Next, we extracted nuclear/cytoplasmic fraction of AGS cells and analyzed ZEB1 subcellular distribution. Compared to uninfected cells, *H. pylori* infection significantly increased steady-state ZEB1 in nucleus and decreased its level in cytoplasm (Fig. 4K). Although MG132 inhibition showed no obvious effect on ZEB1 translocation induced by *H. pylori* infection, steady-state ZEB1 in cytoplasm was significantly increased in uninfected cells. Taken together, these results suggested that *H. pylori* infection promotes deubiquitination and nuclear stabilization of ZEB1.

In addition, *H. pylori* infection also significantly increased the ZEB1-enriched PRTG signal (Fig. 4L) and the luciferase activity of PRTG promoter increased by ZEB1 overexpression (Fig. 4M). Knockdown of ZEB1 expression significantly blocked the increase of PRTG expression induced by *H. pylori* infection (Fig. 4N). These results suggest that ZEB1 is a key mediator for *H. pylori*-mediated upregulation of PRTG, and *H. pylori* infection promotes stabilization and recruitment ZEB1 to PRTG promoter.

### *H. pylori* depends on ZEB1-mediated transcription of PRTG to activate cGMP/PKG pathway

**Fig. 5:**
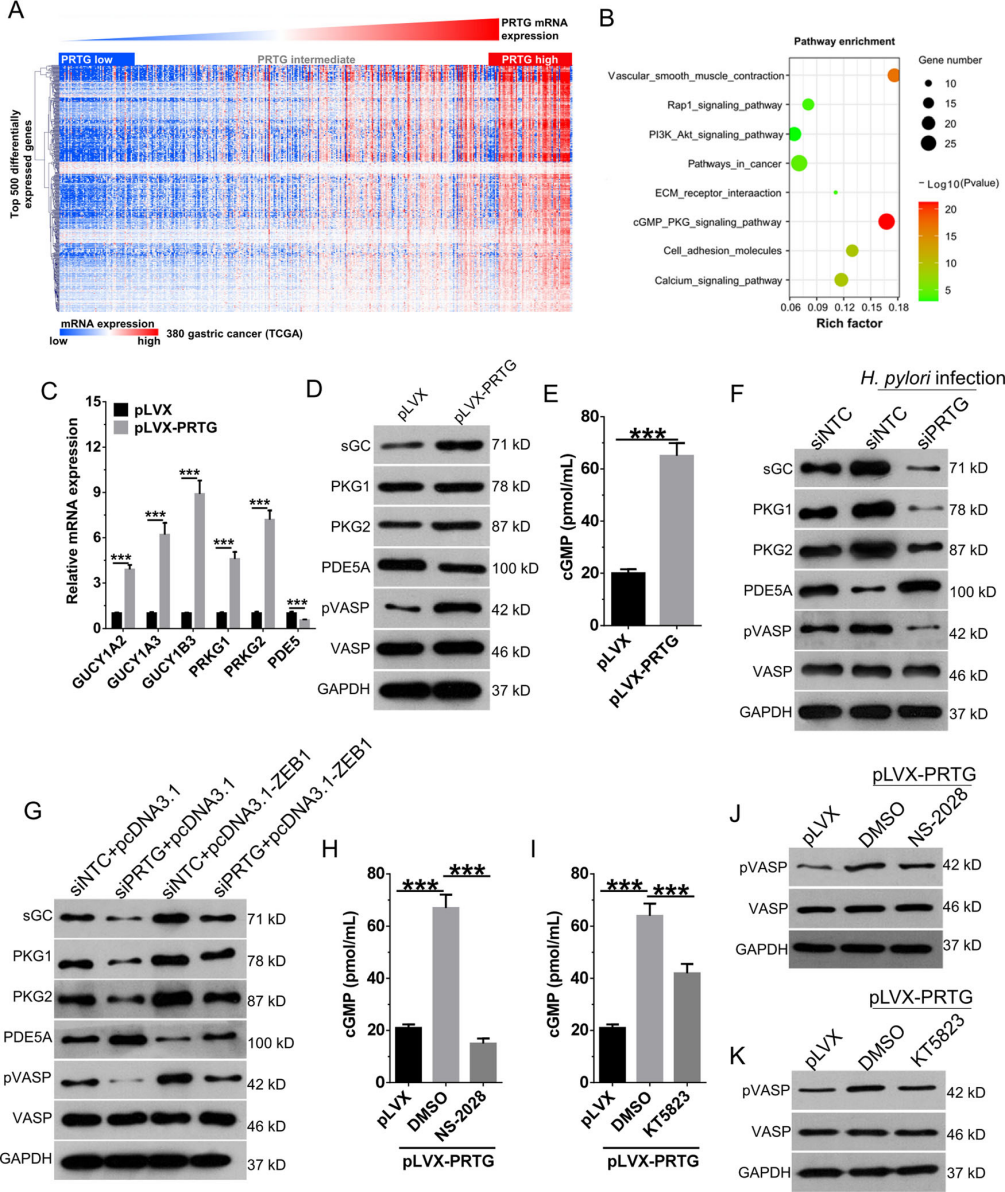
PRTG activates the downstream cGMP/PKG signaling pathway in gastric cancer cells. **A** Expression of top 500 genes positively associated with PRTG expression in gastric patients from TCGA database. **B** Gene ontology term enrichment analysis for top 8 biological process controlled by differentially expressed genes in gastric patients. **C** and **D** The expression of cGMP/PKG signaling pathway related proteins in PRTG-overexpressing AGS cells were detected by qRT-PCR (**C**) and western blot (**D**). **E** Supernatant cGMP levels in PRTG overexpressing AGS cells were detected by ELISA. **F** PRTG silencing AGS cells were infected with *H. pylori* for 48 h, and then the expression of cGMP/PKG signaling pathway related proteins were detected by western blot. **G** AGS cells were transiently co-transfected with ZEB1 overexpressing plasmid and PRTG siRNA. 48 h later, the expression of cGMP/PKG signaling pathway related proteins were detected by western blot. **H**–**K** PRTG overexpressing AGS cells were treated with sGC inhibitor (NS-2028, 10 μM) or PKG inhibitor (KT5823, 1 μM) for 24 h. cGMP levels in supernatant and phosphorylated VASP expression were detected by ELISA and western blot, respectively. Data were presented as mean ± SD from the three independent replicates. ***P < 0.0001.

To further explore the downstream molecular mechanism of PRTG, we selected the top 500 genes positively correlated with PRTG expression in the TCGA database (Fig. 5A) and analyzed signal pathways controlled by these 500 genes. Notably, in addition to those in pathways involved in cancer, genes in the cGMP/PKG signaling pathway were also significantly enriched (Fig. 5B). As predicted, PRTG overexpression led to a significantly elevated mRNA expression of GUCY1A2, GUCY1A3 and GUCY1B3 (mRNA components of sGC enzyme), as well as sGC protein expression (Fig. 5C, D) in gastric cancer cells. The levels of downstream molecules, including cGMP, PKG1 (PRKG1), PKG2 (PRKG2), pVASP were significantly upregulated by PRTG overexpression (Fig. 5C–E). We next tested whether *H. pylori* infection and ZEB1 could activate cGMP/PKG pathway via enhancing PRTG expression. Indeed, *H. pylori* infection and ZEB1 overexpression activated cGMP/PKG pathway in gastric cancer cells, and which can be blocked by the PRTG silencing (Fig. 5F, G). In addition, the addition of sGC inhibitor NS-2028 and PKG inhibitor KT5823 significantly blocked the increase of cGMP levels (Fig. 5H, I) and pVASP levels (Fig. 5J, K) in gastric cancer cells induced by PRTG overexpression. Overall, our results suggest that ZEB1-mediated transcription of PRTG is critical to the activation of cGMP/PKG signaling pathway induced by *H. pylori* infection in gastric cancer.

### PKG inhibitor antagonize the effect of PRTG-induced gastric cancer progression and enhance the effect of chemotherapy

**Fig. 6:**
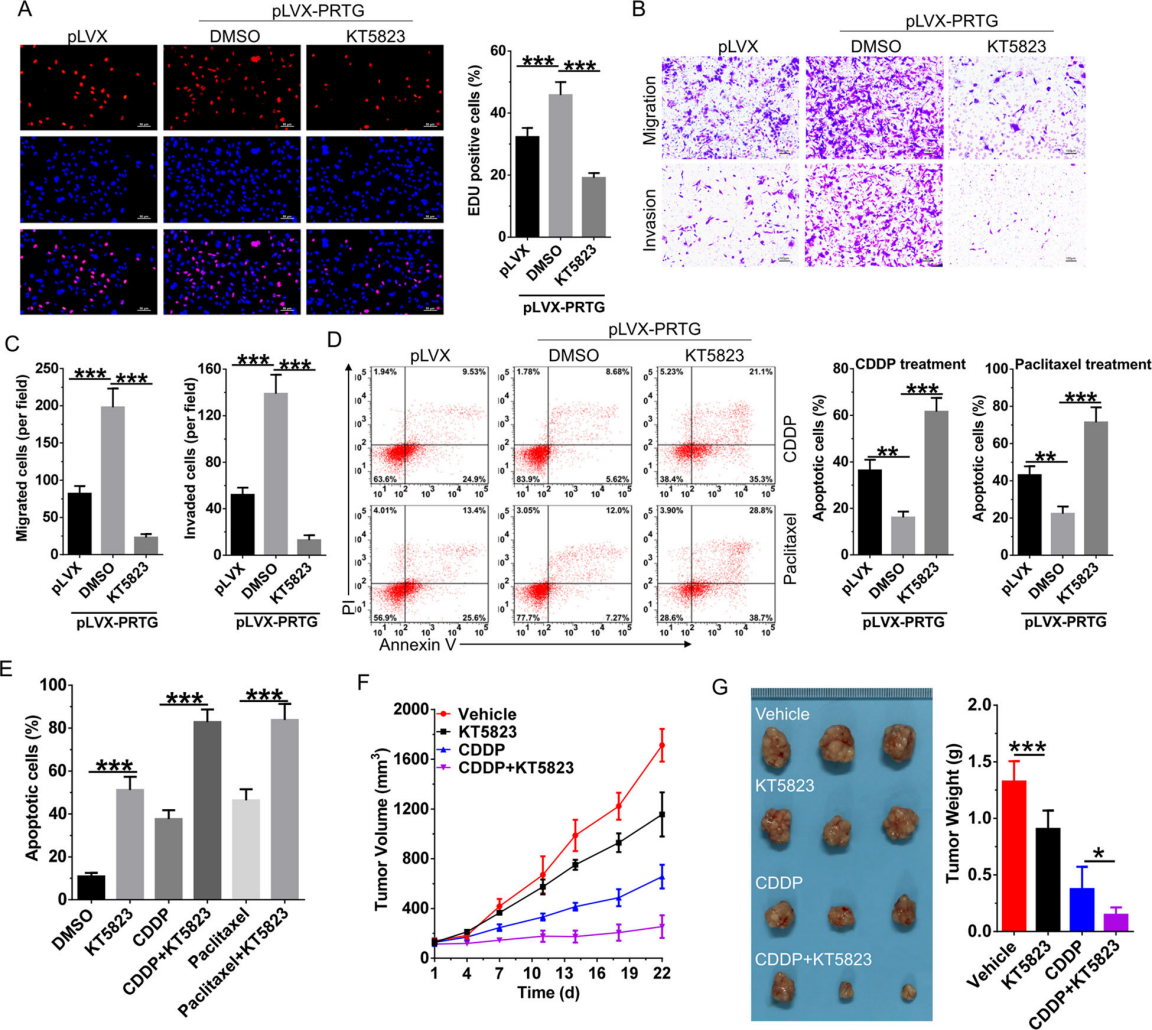
PKG inhibitor antagonizes the effect of PRTG-induced GC progression and enhances the effect of chemotherapy. **A** The EdU was used to determine the proliferation of PRTG-overexpressing AGS cells after treated with PKG inhibitor (KT5823, 1 μM) for 24 h. **B** Migration and invasion of PRTG-overexpressing AGS cells was determined after treated with PKG inhibitor (KT5823, 1 μM) for 24 h. **C** Statistical analysis of migrated and invaded AGS cells (*n* = 3). **D** Apoptosis was detected in PRTG-overexpressing AGS cells after simultaneously treated with PKG inhibitor (KT5823, 1 μM) and chemotherapy drugs (paclitaxel, 20 nM; or CDDP, 5 μM) for 48 h. **E** Flow cytometry was used to examine the synergetic effect of PKG inhibitor (KT5823) and chemotherapy drugs on apoptosis of AGS cells in vitro. **F** In vivo tumor-bearing mouse model (*n* = 12) was used to detect the synergistic effect of PKG inhibitor (KT5823) and CDDP on the growth of AGS cells. **G** Mice were sacrificed, and xenografts tumors were harvested and weighed 22 days after drug treatment, xenografts tumors of 3 mice were presented. Data were presented as mean ± SD from the three independent replicates. *P < 0.05; **P < 0.01; ***P < 0.0001. CDDP, cisplatin.

The function of cGMP/PKG pathway in cancer is tumor- and tissue-specific, and which may be resulted by the sophisticated role of PKG1 and PKG2^19,20^. Thus, we further evaluated the effects of PKG inhibitor KT5823 on PRTG-mediated tumorigenic activities. In gastric cancer cells, PKG inhibitor KT5823 treatment significantly reversed the promotive effects of PRTG on proliferation (Fig. 6A), invasion and migration (Fig. 6B, C). Furthermore, KT5823 also blocked the chemoresistant effects of PRTG in gastric cancer cells in response to CDDP and paclitaxel, as reflected by enhanced apoptosis (Fig. 6D) and pH2AX foci formation (Fig. S5A). Thus, we further evaluated the synergistic effects of KT5823 with chemotherapeutic drugs. As predicted, KT5823 treatment significantly enhanced the pro-apoptotic effects of CDDP and paclitaxel in gastric cancer cells (Figs. 6E and S5B). Next, the synergistic effect of KT5823 with CDDP was further tested in in vivo tumor-bearing nude mice. Our results showed that tumor volumes in mice treated with KT5823 and CDDP grew significantly slower than either KT5823 or CDDP treatment alone (Fig. 6F). Mice were euthanized 21-days post-treatment and a considerably lower volume and weight of tumors were observed in mice treated with KT5823 and CDDP (Fig. 6G). Taken together, these data demonstrate a requirement for cGMP/PKG pathway activation in PRTG-mediated oncogenic activities and supports PKG inhibition as a viable therapeutic strategy against gastric cancer.

## Discussion

To unveil the potential pathogenic mechanisms of *H. pylori* contributed to the progression of gastric cancer, several integrative analyses have investigated the dysregulated lncRNA/mRNA/miRNA expression profiles in gastric epithelial cells (GES-1 cells/AGS cells/clinical specimens) in response to *H. pylori* infection^11,21,22^. However, the expression status and roles of these dysregulated genes that associated with *H. pylori* infection in the progression of gastric cancer remain largely unknown. In this study, by comparing mRNA expression profile associated with both gastric cancer progression and *H. pylori* infection, we have identified a novel oncogenic role of *H. pylori-*upregulated PRTG in gastric cancer.

PRTG has been proposed to involve in nervous system development via enhancing the migration and survival of EMT-like cephalic neural crest cells, and mediate the pathogenesis of osteoarthritis^12,23,24^. In the present study, overexpression of PRTG enhances survival, metastatic and chemoresistant potential in gastric cancer cells both in vitro and in vivo. Therefore, our study firstly reveals the possibility that PRTG acts as an oncogenic protein in cancer cells. To further unveil the molecular mechanisms of how *H. pylori* regulated PRTG expression, we identified a candidate list of transcriptional regulators of TFs predicted to bind the PRTG promoter and demonstrated that ZEB1 is a positive regulator and directly enhances PRTG expression via binding to the −469 to −459 promoter region.

ZEB1 drives EMT and confers chemotherapeutic resistance of cancer cells via directly altering the expression of a plethora of genes, like ESRP1 and ATM^25,26^. In this study, our data clearly demonstrate that PRTG is a novel transcriptional target of ZEB1. Recently, Baud et al.^27^ reported that *H. pylori* initiates a mesenchymal transition through ZEB1 in gastric epithelial cells. Moreover, in our present study, *H. pylori* promotes deubiquitination and stabilization of ZEB1 in nucleus, and which result in the prolonged binding between ZEB1 and PRTG promoter. Importantly, knockdown of ZEB1 significantly blocked *H. pylori*-mediated PRTG upregulation. These results indicate that ZEB1 is an important mediator in *H. pylori*-associated gastric cancer.

Different from Baud et al.^27^ and Sougleri et al.^28^ reported that *H. pylori* increases transcriptional level of ZEB1 in an CagA-dependent manner in *H. pylori* 26695 strain and clinical isolates, treatment of AGS cells with CagA^+^ *H. pylori* strain ATCC43504 showed no significant impact on ZEB1 mRNA expression in this study. Thus, we speculate that the regulation mechanism of ZEB1 expression may be *H. pylori* strain context-dependent. As a master EMT-inducing transcription factor that plays essential role in tumor invasion, metastasis, and therapy resistance, ZEB1 can be degraded by ubiquitin ligase Siah1/2 and Skp1-Pam-Fbxo45 complex in an ubiquitination-proteasome-dependent manner, and stabilized by FLICE/caspase-8-associated huge protein (FLASH) and deubiquitinases USP51^17,29^. Thus, further studies are needed to more fully elucidate the molecular mechanisms involved in *H. pylori*-induced ZEB1 stabilization or transcription.

Although we demonstrate that PRTG plays important oncogenic role in *H. pylori*-associated gastric cancer, but a detailed understanding of how PRTG contributes to gastric cancer progression is still lacking. To solve this question, we analyzed the signal pathways controlled by the top 500 genes positively correlated with PRTG expression in the TCGA database. Intriguingly, multiple members of the cGMP/PKG signaling pathway were positively associated with PRTG expression. Indeed, our data demonstrate that the cGMP/PKG signaling-associated proteins, including sGC, PKG1/2 and pVASP were significantly upregulated by PRTG in gastric cancer cells. Moreover, *H. pylori* and ZEB1 were depended on PRTG to activate cGMP/PKG signaling pathway. The major effectors of cGMP/PKG signaling pathway, including three serine-threonine kinases, PKG1α, PKG1β and PKG2, which different in their N-terminal domains and thus have distinct subcellular localization and substrate specificity^30^. PKG1α promotes chemoresistance, maintenance of cancer stem cells and cell survival in lung, cervical, breast and ovarian cancers^19,31–33^; while overexpression of PKG1β and PKG2 exerts anti-tumorigenic effects in head and neck squamous cell carcinoma, breast, prostate and colon cancers^20,34–36^. Different from the loss of PKG1α in head and neck squamous cell carcinoma cells^20^, PKG1α and PKG2 expressed in gastric cancer cells, and were upregulated by *H. pylori* via ZEB1/PRTG axis. Thus, PKG may play a sophisticated role in the progression of gastric cancer and require further elucidation.

p-VASP^Ser239^ is an indicator of PKG1α activation^37^, and importantly, it also increased in PRTG-overexpressing cells and could be blocked by inhibitors target to sGC and PKG. Since our present data suggest that PRTG functions as a oncogenic protein in gastric cancer and the downstream VASP also drives cell proliferation and metastasis in cancer cells^38,39^, it was logical to suppose that antagonists of PKG such as KT5823 (target to PKG1/2) could have the potential to efficiently block the oncogenic function of PRTG and provide a potential therapeutic avenue to treat gastric cancer. Indeed, KT5823 treatment significantly antagonized the tumorigenic effects of PRTG and further enhanced the effect of chemotherapy both in vitro and in vivo. In addition, KT5823 also enhances sildenafil-induced apoptosis in colorectal cancer^40^ and reduces the maintenance of cancer stem cells in breast cancer^32^. Owing to the lack of suitable blockers target to ZEB1/PRTG axis, the development of more specific and pharmacologically tractable antagonists that block PKG1α activity could provide novel approaches to improve gastric cancer outcomes.

**Fig. 7:**
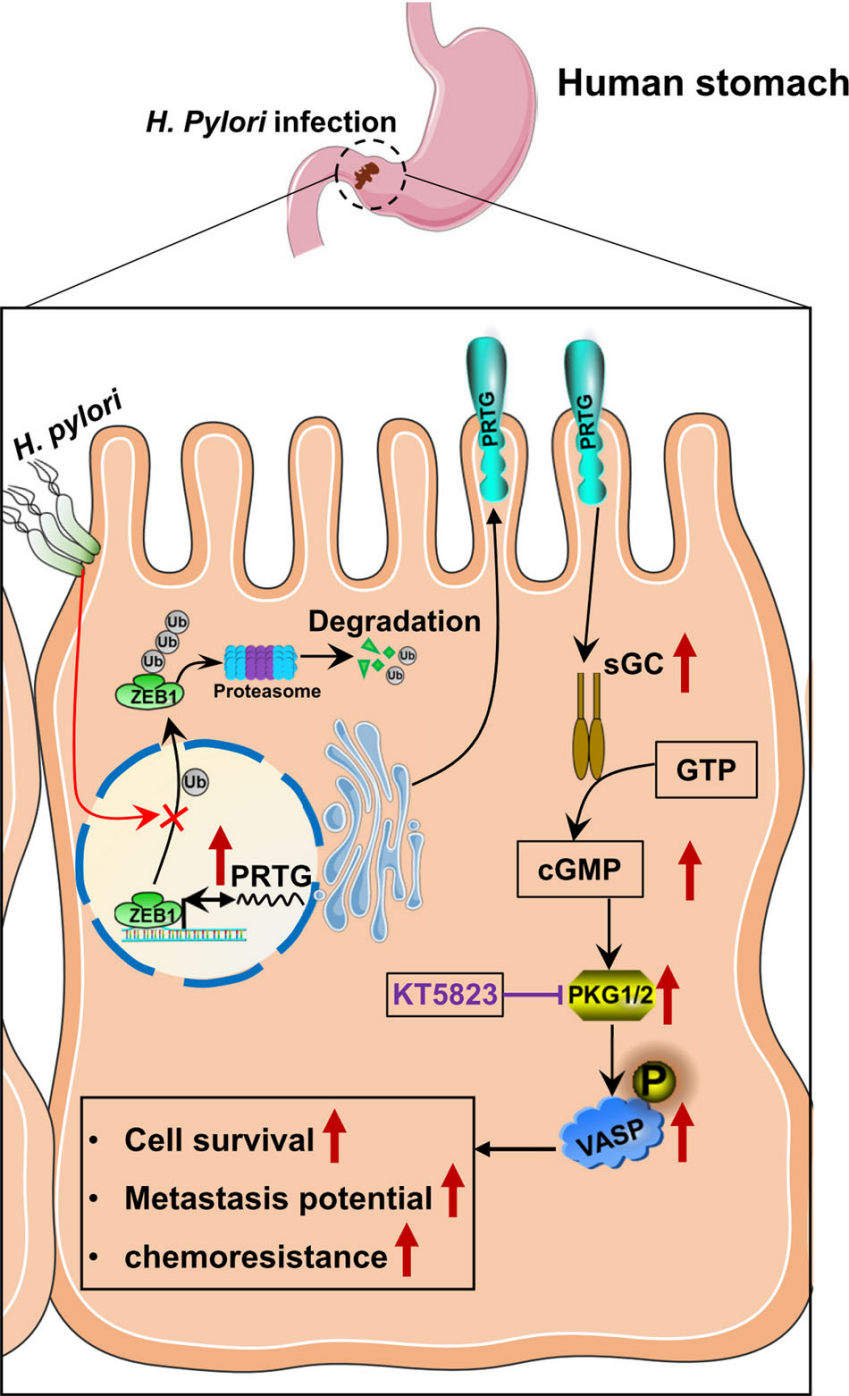
Schematic diagram of the ZEB1/PRTG/cGMP/PKG regulatory mechanism in *H. pylori*-infected gastric cells. *H. pylori* promotes ZEB1 stabilization and recruitment to PRTG promoter, and which resulted the upregulation of PRTG expression. Then, PRTG activated the subsequent cGMP/PKG signaling pathway to perform oncogenic activities. PKG inhibitor KT5823 antagonizes the effect of PRTG-induced gastric cancer progression and enhance the effect of chemotherapy.

In summary, we have demonstrated that PRTG is a novel oncogenic protein that plays important role in *H. pylori-induced* gastric carcinogenesis by activating the downstream cGMP/PKG signaling pathway. *H. pylori* infection enhances PRTG transcription by promoting ZEB1 stabilization and recruitment to the PRTG promoter. Therefore, our findings provide new insights into the molecular mechanisms involved in *H. pylori*-mediated gastric cancer progression. Several drugs directly target to cGMP/PKG pathway have been clinically approved for treating non-malignant conditions. Thus, blocking PRTG activation through compounds specifically target to cGMP-PKG signaling pathway could provide a novel therapeutic approach to treat gastric cancer (Fig. 7).

## Supplementary information

Supplementary Figure legends

Figure S1

Figure S2

Figure S3

Figure S4

Figure S5

Supplementary Table
