# Supplementary material for: The novel ZEB1-upregulated protein PRTG induced by Helicobacter pylori infection promotes gastric carcinogenesis through the cGMP/PKG signaling pathway
Source: Cell Death Dis. 2021 Feb 4;12(2):150. doi: 10.1038/s41419-021-03440-1 (PMC7862680; doi:10.1038/s41419-021-03440-1)
Supplement: Supplementary file 1 — Supplementary Figure legends [file 41419_2021_3440_MOESM1_ESM.docx]

**Supplementary Figure legends**

**Figure S1. PRTG plays oncogenic activities in gastric cancer progression (related to Figure 2). A** Western blot analysis of the expression level of PRTG in gastric cancer and adjacent normal tissues. **B** Western blot was used to determine the expression level of PRTG in gastric cancer cell lines with different malignant potential. **C** Western blot was used to determine the overexpressing and interfering efficiency of PRTG in AGS cells. **D** CCK-8 assay was used to detect the effect of PRTG on the cellular cytotoxicity of chemotherapy drugs (paclitaxel, 20 nM; or CDDP, 5 μM) in AGS cells. **E** Statistical analysis of cell cycle distribution in PRTG overexpressing or silencing AGS cells after treated with chemotherapy drugs (paclitaxel, 20nM; CDDP, 5 μM) for 48 h. **F** Cell cycle distribution in PRTG overexpressing or silencing AGS cells after treated with chemotherapy drugs (paclitaxel, 20nM; CDDP, 5 μM) for 48 h was detected by flow cytometry. **G** Immunofluorescence detection of the effect of PRTG on the expression of DNA damage repair marker pH2AX in paclitaxel-treated AGS cells. **H** Statistical analysis of the invasion and migration ability of PRTG overexpressing or silencing AGS cells related to Figure 2E. **I** Western blot analyses were used to detect the effect of PRTG on EMT markers in AGS cells. Data were presented as mean ± SD from the three independent replicates. ***P*<0.01; ****P*<0.0001. Abbreviation: CDDP, cisplatin; EMT, Epithelial to Mesenchymal Transition.

**Figure S2.** **PRTG plays oncogenic activities in gastric cancer MGC-803 cells. A** PRTG overexpression (stable cell line acquired by lentivirus infection) and knockdown (siRNA transient transfection) efficiency in MGC-803 cells were monitored before conducting cellular studies. **B** Cellular proliferation after PRTG stable overexpression or transient knockdown in MGC-803 cells were detected by EdU assay. **C** Cellular apoptosis was detected in PRTG overexpressing or silencing MGC-803 cells after treated with chemotherapy drugs (paclitaxel, 20 nM; CDDP, 5 μM) for 48 h. **D** Transwell assay was used to detect the invasion and migration ability of PRTG overexpressing or silencing MGC-803 cells. Data were presented as mean ± SD from the three independent replicates. **P*<0.05; ****P*<0.0001. Abbreviation: CDDP, cisplatin; pLVX, empty control pLVX lentivirus.

**Figure S3. PRTG is required for *H. pylori* to promote GC progression. A** IHC staining of PRTG expression in AGS cells after infected with *H. pylori* (MOI=10:1) for 48 h. **B** The expression of PRTG in PRTG-silencing AGS cells after infected with *H. pylori* (MOI=10:1) for 48 h. **C** Statistical analysis of the proliferation of PRTG-silencing AGS cells after infected with *H. pylori* (MOI=10:1) for 48 h related to Figure 3C. **D** Cell cycle distribution was detected in PRTG silencing AGS cells after treated with chemotherapy drugs (paclitaxel, 20nM; CDDP, 5 μM) and infected with *H. pylori* (MOI=10:1) for 48 h. **E** Statistical analysis of cell cycle distribution in D (n=3). **F** Immunofluorescence detection of the effect of PRTG on the expression of DNA damage repair marker pH2AX in AGS cells after treated with paclitaxel (20nM) and infected with *H. pylori* (MOI=10:1) for 48 h. Data were presented as mean ± SD from the three independent replicates. ****P*<0.0001. Abbreviation: CDDP, cisplatin.

**Figure S4. ZEB1 overexpression predicts poor prognosis of gastric cancer.** **A** Positive association between PRTG and ZEB1 expression in 152 local gastric cancer tissues was confirmed by spearman rank correlation analysis. **B** qRT-PCR was used to detect the expression of ZEB1 mRNA in gastric cancer and adjacent normal tissues from local hospital. Boxes represent medians and interquartile ranges, with whiskers indicating 10% to 90% range. Dots represent patients who fell outside the 10% to 90% range. **C** Kaplan-Meier survival plot analysis of the overall survival for 152 gastric cancer patients in local hospital with high or low ZEB1 expression. **D** Kaplan-Meier survival plot analysis of the overall survival in gastric cancer patients from GEO dataset GSE62254 and TCGA database with high or low ZEB1 expression. **E** qRT-PCR was used to detect the expression of ZEB1 in *H. pylori^+^* or *H. pylori^-^* gastric cancer tissues from local hospital. Boxes represent medians and interquartile ranges, with whiskers indicating 10% to 90% range. Dots represent patients who fell outside the 10% to 90% range. **F** The mRNA expression of ZEB1 in AGS cells at different time points post *H. pylori* infection was detected by qRT-PCR. **G** The mRNA expression of ZEB1 in MGC-803 cells at different time points post *H. pylori* infection was detected by qRT-PCR. **H** The expression of ZEB1 in MGC-803 cells at different time points post *H. pylori* infection was detected by western blot. Abbreviation: OS, overall survival.

**Figure S5. PKG inhibitor and chemotherapy drugs have synergistic effects in gastric cancer (related to Figure 6).** **A** Immunofluorescence detection of pH2AX expression in PRTG-overexpressing AGS cells after simultaneously treated with PKG inhibitor (KT5823, 1 μM) and paclitaxel (20nM) for 48 h. **B** Flow cytometry was used to examine the synergetic effect of PKG inhibitor (KT5823) and chemotherapy drugs on apoptosis of AGS cells *in vitro* (related to Figure 6E). Abbreviation: CDDP, cisplatin.
