# Supplementary figures and images for: The novel ZEB1-upregulated protein PRTG induced by Helicobacter pylori infection promotes gastric carcinogenesis through the cGMP/PKG signaling pathway
Source: Cell Death Dis. 2021 Feb 4;12(2):150. doi: 10.1038/s41419-021-03440-1 (PMC7862680; doi:10.1038/s41419-021-03440-1)

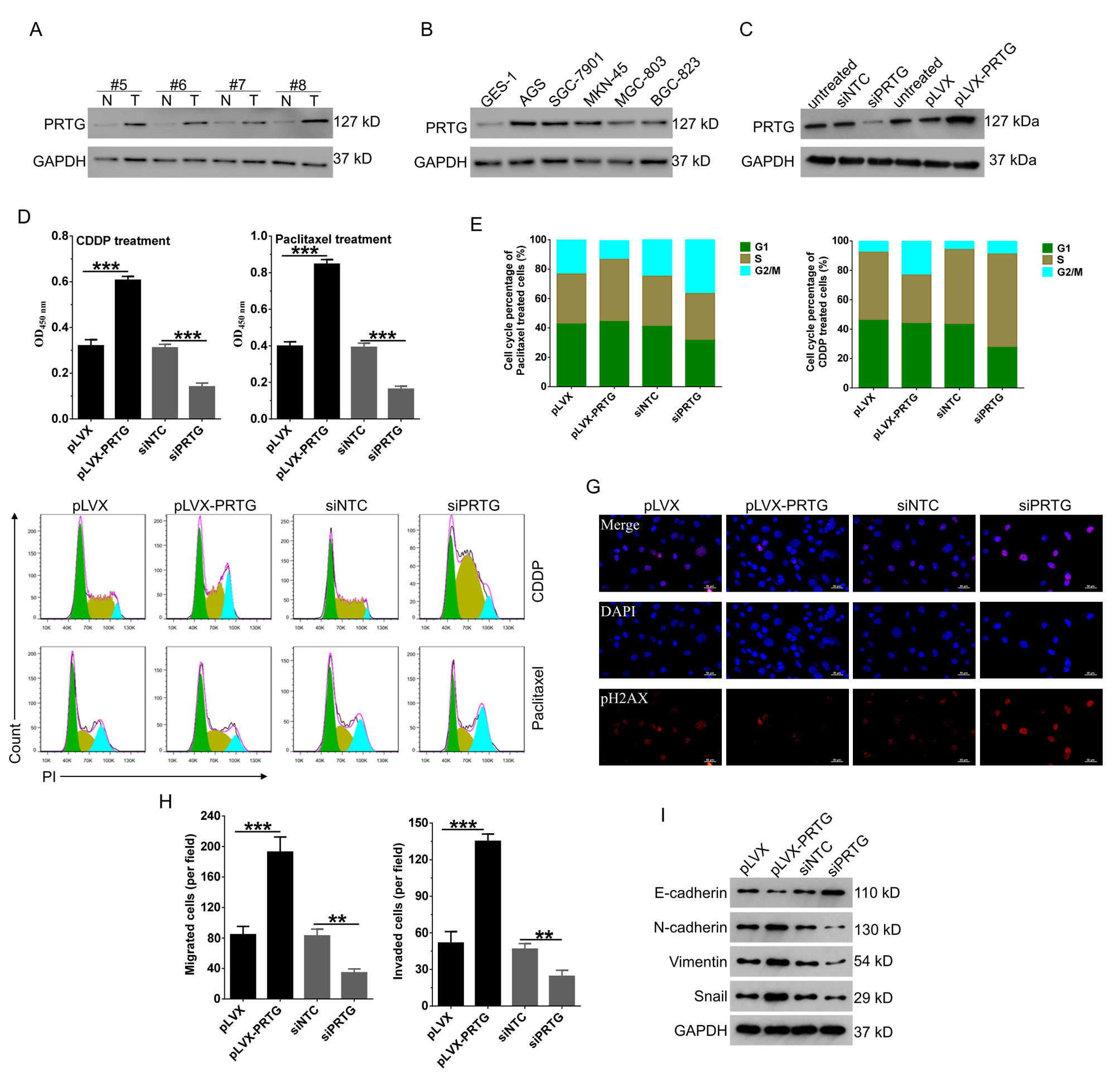

Supplement: Supplementary file 2 — Figure S1 [file 41419_2021_3440_MOESM2_ESM.tif]

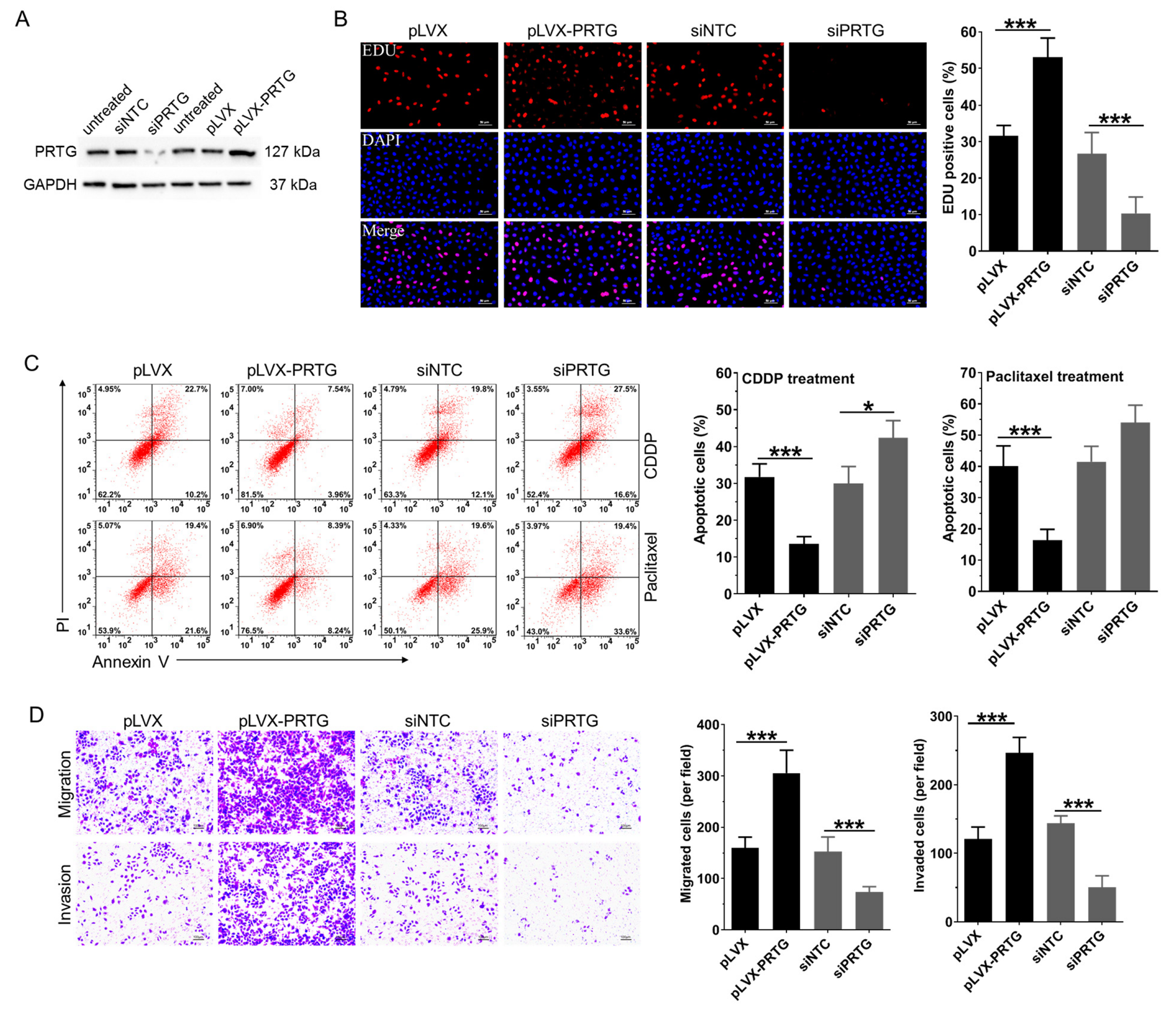

Supplement: Supplementary file 3 — Figure S2 [file 41419_2021_3440_MOESM3_ESM.tif]

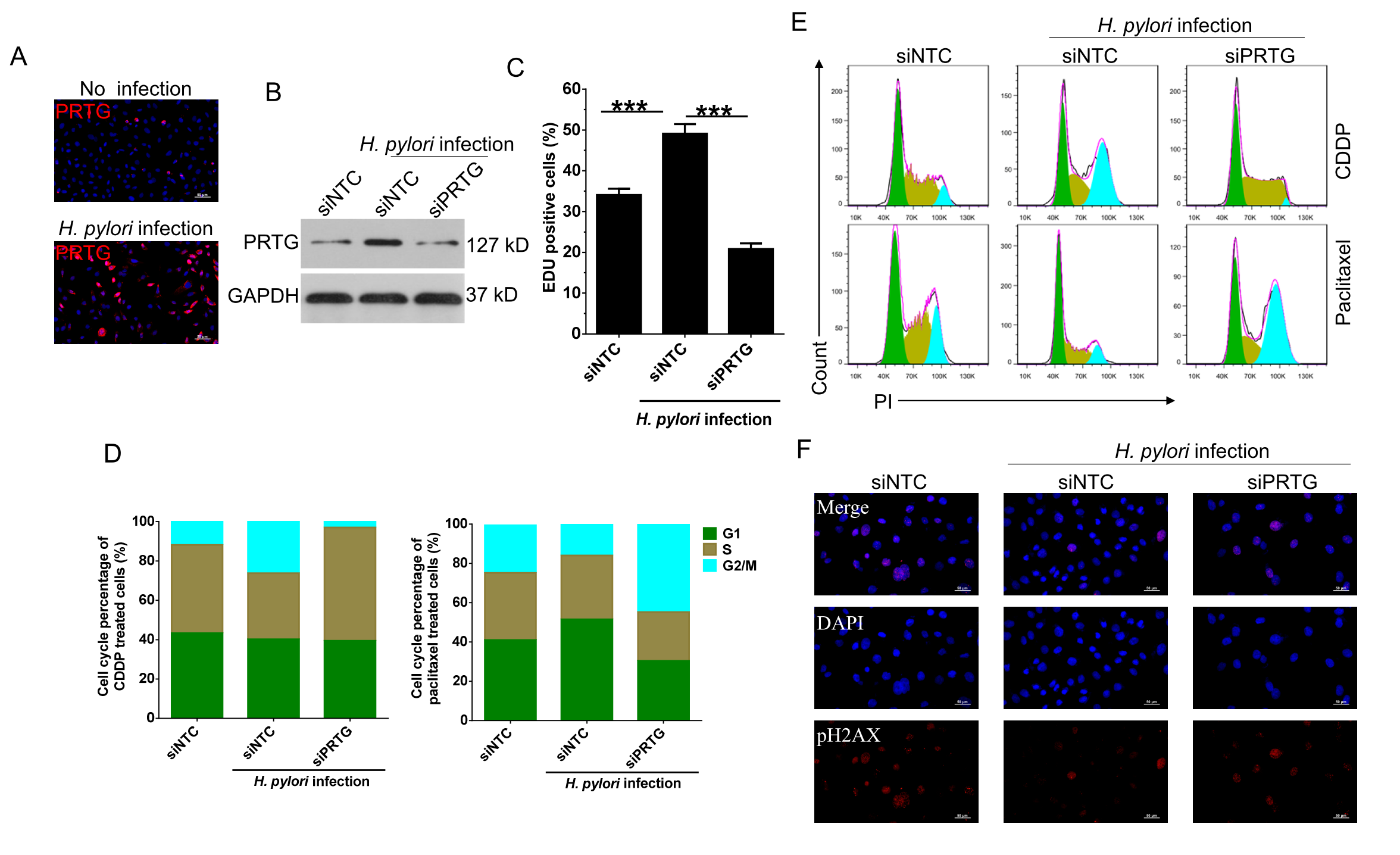

Supplement: Supplementary file 4 — Figure S3 [file 41419_2021_3440_MOESM4_ESM.tif]

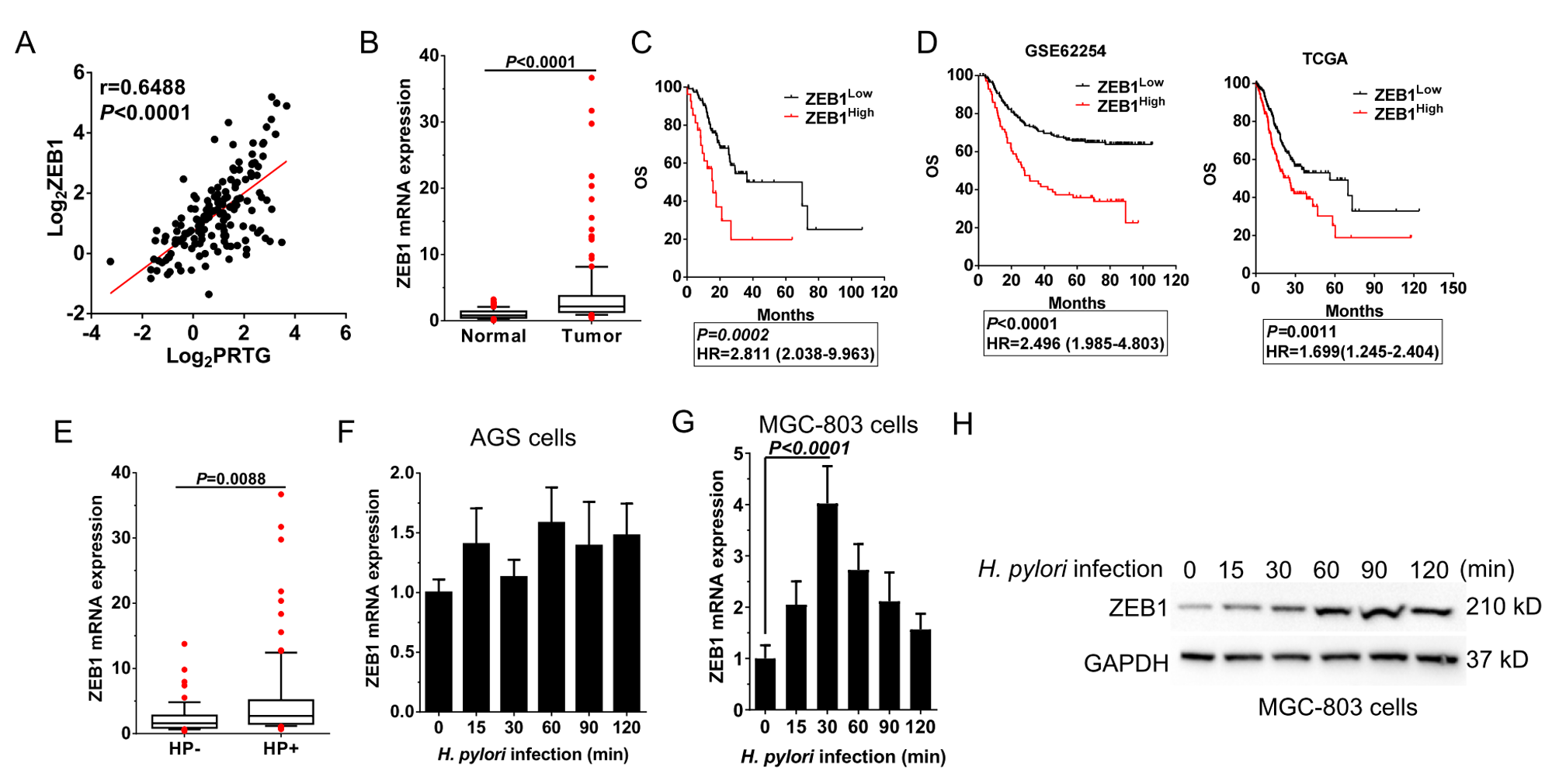

Supplement: Supplementary file 5 — Figure S4 [file 41419_2021_3440_MOESM5_ESM.tif]

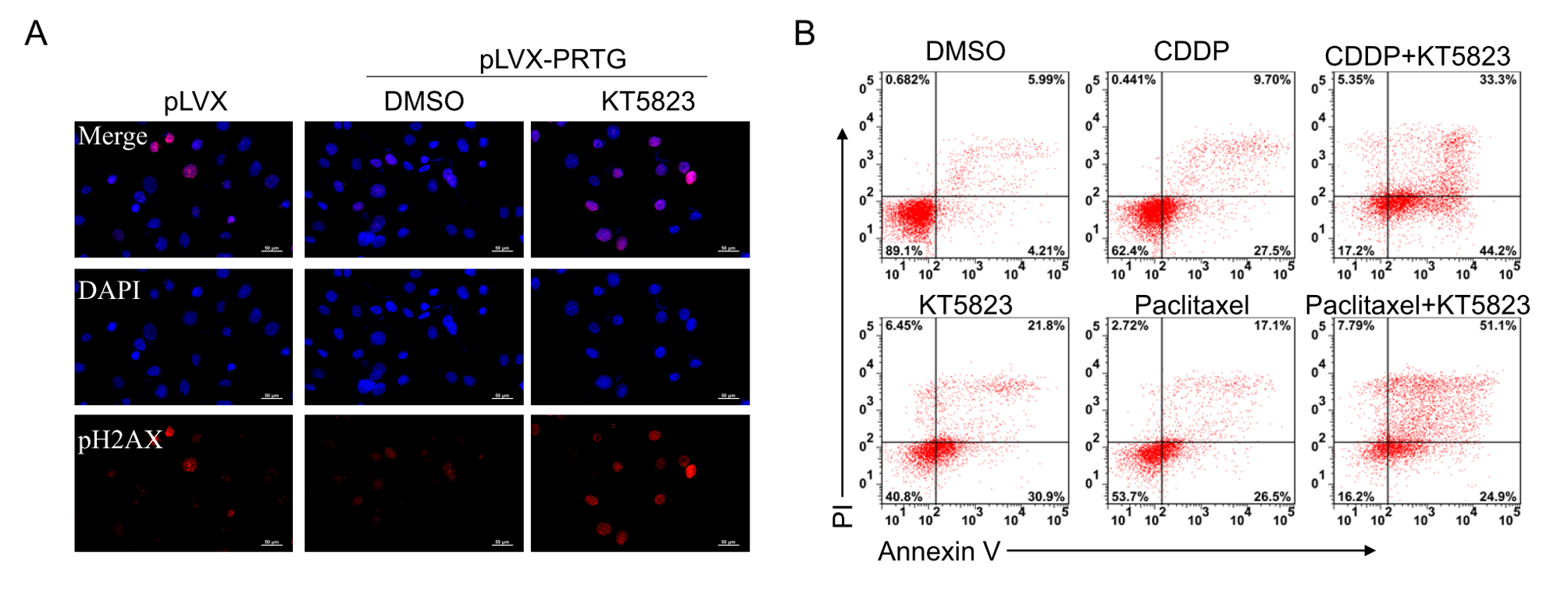

Supplement: Supplementary file 6 — Figure S5 [file 41419_2021_3440_MOESM6_ESM.tif]
