# Supplementary material for: The novel ZEB1-upregulated protein PRTG induced by Helicobacter pylori infection promotes gastric carcinogenesis through the cGMP/PKG signaling pathway
Source: Cell Death Dis. 2021 Feb 4;12(2):150. doi: 10.1038/s41419-021-03440-1 (PMC7862680; doi:10.1038/s41419-021-03440-1)
Supplement: Supplementary file 7 — Supplementary Table [file 41419_2021_3440_MOESM7_ESM.docx]

**Supplementary Table 1. Sequence of primers**

| Gene construct | Gene | Forward | Reverse |
| --- | --- | --- | --- |
| mRNA qPCR | | | |
|  | PRTG | TGCATGCAAGATTCATCCCACCC | TGCAATACTCCTGTTGGTAGGGCA |
|  | GAPDH | GATCATCAGCAATGCCTCCT | TGTGGTCATGAGTCCTTCCA |
|  | GUCY1A2 | AGTGAAACGGATTTGGGTACTGA | TGGACCAGTCCTTACCTCCA |
|  | GUCY1A3 | ACGAGTGAGGAGATGGGACA | GGTAGAGCCCTCGTCCTGTA |
|  | GUCY1B3 | ACACACTGCTGGGATACTGC | AGAGCTAGAGGCCAATTATTCCA |
|  | PDE5A | TTGCCTAGCAGTGAAGTGCC | CAATTGCTTGTGATGGCCTGT |
|  | PRKG1(α) | CGAGTACTTAGCGCCCATTCA | CCGCTTCTCCAGCTCTTTGAT |
|  | PRKG2 | ATCCCAACTCCACCATTTCCT | GTACAATTCTGCATACAGATACCAA |
|  | EP300 | AAAAATAAGAGCAGCCTGAG | AGACCTCTTTATGCTTCTTCC |
|  | SMARCA4 | AGTGCTGCTGTTCTGCCAAAT | GGCTCGTTGAAGGTTTTCAG |
|  | FOXO1 | TACGAGTGGATGGTCAAGAG | ATGAACTTGCTGTGTAGGGAC |
|  | HSF1 | TGAAAAGTGCCTCAGCGTAGCC | TGCTCAGCATGGTCTGCAGGTT |
|  | NR3C1 | GAGCAGTGGAAGGACAGCAC | CCTGTAGTGGCCTGCTGAAT |
|  | PBX3 | CAAGTCGGAGCCAATGTG | ATGTAGCTCAGGGAAAAGTG |
|  | ZEB1 | ATGTGGCTAGTTTGTCCTC | AGCAAGATTTCCTCCAGGTC |
|  | E2F1 | TGCAGAGCAGATGGTTATGG | AGATGATGGTGGTGGTGACA |
| ChIP qPCR | | | |
|  | GAPDH | TTGTCAACCTTGGGGCAGG | CGCTTCCGGTACTTCTCACG |
|  | P1 of PRTG | CAAGATACTCTGCTGGATGG | TTCTCCCTGTTCCCTTTT |
|  | P2 of PRTG | ATGTGCCACAGATTCAGTTT | GCAGGCTTTGATAGATTACTT |
|  | P3 of PRTG | AGAGCCGTGCCCAAATGC | CCTCCTACACCCTCCTACC |
|  | P4 of PRTG | GACCAGCCGCAGAGCAGA | AGCAGCAGGAGCAGGAGC |
| siRNA | | | |
|  | siNTC | CAGAGGATGGACAGAACACAGTTTA | |
|  | siEP300 | CACAATACCTCGTGATGCCACTTAT | |
|  | siSMARCA4 | CAATTCCAGCGTGGCCTTCAGTTCT | |
|  | siFOXO1 | CCAGATGCCTATACAACA | |
|  | siHSF1 | GAAGACATAAAGATCCGCCAGGACA | |
|  | siNR3C1 | CAGTGGAAGGTAGACAGCACAATTA | |
|  | siPBX3 | CAGTGATGGCCTTGGAGGAAATTCA | |
|  | siZEB1 | TGATCAGCCTCAATCTGCA | |
|  | siE2F1 | CCGTGGACTCTTCGGAGAACTTTCA | |
|  | siPRTG | CCAGGTCAGACAACCAGCTTCTCAA | |
